# Trade-offs constrain the success of glyphosate-free farming

**DOI:** 10.1038/s41598-024-58183-8

**Published:** 2024-04-05

**Authors:** H. Metcalfe, J. Storkey, R. Hull, J. M. Bullock, A. Whitmore, R. T. Sharp, A. E. Milne

**Affiliations:** 1 Net Zero & Resilient Farming, Rothamsted Research, Harpenden, AL5 2JQ UK; 2 Protecting Crops and the Environment, Rothamsted Research, Harpenden, AL5 2JQ UK; 3 UK Centre for Ecology & Hydrology, Wallingford, OX10 8BB UK

**Keywords:** Agroecology, Ecology, Environmental sciences, Computational biology and bioinformatics, Computational models, Ecology, Agroecology, Ecological modelling

## Abstract

Glyphosate, the most widely used herbicide, is linked with environmental harm and there is a drive to replace it in agricultural systems. We model the impacts of discontinuing glyphosate use and replacing it with cultural control methods. We simulate winter wheat arable systems reliant on glyphosate and typical in northwest Europe. Removing glyphosate was projected to increase weed abundance, herbicide risk to the environment, and arable plant diversity and decrease food production. Weed communities with evolved resistance to non-glyphosate herbicides were not projected to be disproportionately affected by removing glyphosate, despite the lack of alternative herbicidal control options. Crop rotations with more spring cereals or grass leys for weed control increased arable plant diversity. Stale seedbed techniques such as delayed drilling and choosing ploughing instead of minimum tillage had varying effects on weed abundance, food production, and profitability. Ploughing was the most effective alternative to glyphosate for long-term weed control while maintaining production and profit. Our findings emphasize the need for careful consideration of trade-offs arising in scenarios where glyphosate is removed. Integrated Weed Management (IWM) with more use of cultural control methods offers the potential to reduce chemical use but is sensitive to seasonal variability and can incur negative environmental and economic impacts.

## Introduction

Glyphosate, the world’s most widely used herbicide^1^, is the subject of widespread controversy in scientific literature, media, policy, and society. The debate revolves around its prominent role in modern agriculture for controlling arable weeds, despite its potential harmfulness and environmental impact. This article aims to assess the trade-offs associated with decreasing glyphosate use and explore the potential for alternative farming strategies using cultural control methods as part of their integrated weed management (IWM)^2^, that could facilitate a transition towards a glyphosate-free future.

Glyphosate's extensive use is primarily attributed to its application in glyphosate-resistant crops, particularly in the Western Hemisphere^3^. Yet even in countries where the use of such crops is prohibited, glyphosate remains widely employed. For instance, a 2018 survey revealed that glyphosate accounted for 17% of the herbicide-treated area in the UK, equating to almost 2466 tonnes of active substance applied^4^. The widespread use of glyphosate outside of glyphosate-resistant genetically modified (GM) crops can be attributed to its broad-spectrum efficacy—meaning it controls both broadleaved weeds and grasses. The primary use of glyphosate in arable cropping systems is to control weeds during the fallow period between crops, where it is applied before sowing or crop emergence to reduce the weed burden for the subsequent crop. It is also used to kill cover crops and temporary grass leys prior to the cultivation of annual crops. Finally, glyphosate can be applied as a desiccant in some combine-harvested crops before harvest. The importance of glyphosate for weed control in stubbles (in the absence of tillage) and the management of cover crops and leys make it a central tool in the adoption of conservation or regenerative agriculture that focus on improving soil health^5,6^. However, the environmental and health impacts associated with glyphosate may trade-off against some of the benefits of moving to more sustainable systems that reduce tillage and integrate cover crops^6^.

Despite glyphosate’s extensive use, concerns have been raised regarding its potential negative impacts on human health^7^. The detection of glyphosate residues on various food items^8^ has fuelled the debate, with a particular focus on its probable carcinogenic risk, as identified by the World Health Organisation^9^. However, experts remain divided on this issue with some suggesting the risk is low due to the levels of exposure required for this to be a threat^10,11^. Persistence in soil and water presents additional environmental concerns^12^ and there is strong evidence suggesting that typical glyphosate concentrations in freshwater pose a moderate to high risk to aquatic organisms^13^. A third area of concern is the evolution of herbicide resistance to glyphosate. Glyphosate resistance was first noted in 1996 in an Australian population of *Lolium rigidium* and since then it has been reported in thirty-eight weed species^14^—primarily in farming systems where glyphosate-resistant crops are used. While not currently a significant problem in European arable cropping systems, there is evidence for declining sensitivity of weeds to glyphosate, related to frequency of use^15^, and glyphosate resistance has been confirmed in a UK population of *Bromus sterilis*^16^*,* a grass weed that is adapted to reduced tillage systems. It is, therefore, prudent from an agronomic (as well as regulatory) perspective to explore alternatives to reduce its use and consequently selection pressure for evolved resistance.

Considering these concerns around human health, environmental impact and the potential for resistance evolution, several countries have already banned the use of glyphosate. While the US and the EU have not implemented a complete ban, certain restrictions have been imposed, and discussions regarding a potential phase-out are underway^17^. However, some growers strongly oppose such changes, as they argue that banning glyphosate could undermine current agricultural best practices and potentially threaten food security^18^. Consequently, there is increasing interest in identifying alternative weed control methods that are less damaging to the environment and human health. Understanding the potential impacts of ceasing glyphosate use and transitioning to alternative strategies is therefore essential for the scientific community, farmers, policymakers, and the public.

This study aimed to address the research gap by comprehensively assessing the potential impacts of discontinuing glyphosate use. We explored the implementation of three important IWM practices^2^ to mitigate any adverse effects of glyphosate withdrawal. These were: (I) the diversification of crop rotations (either by increasing the frequency of spring cropping or grass leys to disrupt weed life cycles), (II) the delayed drilling of autumn crops (to control early emerging cohorts through cultivation) and (III) the use of inversion ploughing (to bury weed seeds). We kept the use of all other management practices, including the use of a typical pre- and post-emergence herbicide program constant. We tested three hypotheses related to the replacement of glyphosate with IWM practices: (1) Loss of glyphosate will lower production and profits, increase weed abundance and arable plant diversity, but decrease the risk to the environment associated with herbicides; (2) The impacts of glyphosate loss on production will be greatest in farms with high levels of weed resistance to other herbicides; (3) The impacts of glyphosate loss can be mitigated through the implementation of IWM practices.

We utilized a process-based model to evaluate the effects of these alternative practices on productivity, profit, weed abundance, arable plant diversity, and herbicide risk to the environment. By simulating a typical winter wheat focussed arable farming system that relies on glyphosate but lacks glyphosate-resistant crops, we assessed the consequences of removing glyphosate and explored the alternative practices described above. By starting with realistic cropping rotations, weed pressure and weather patterns based on empirical data for current practices and environments, our approach simulates potential trajectories of adaptation to a loss of glyphosate.

## Methods

We used the Rothamsted Landscape Model (RLM)^19^ to simulate potential future scenarios with and without glyphosate. RLM simulates the growth of arable crops as well as soil processes such as water and nutrient flows. RLM also includes a non-crop plant component^20^, which has been calibrated to show functional changes in weed communities. These weed communities compete with the crop for light and there is a feedback mechanism by which the amount of light available to the crop is modified, reducing its growth rate. Plant growth can be limited by low levels of nitrogen in species which require particularly elevated levels of nitrogen, but there is no competition for nutrients between weeds and crops. This is typically the case in conventionally managed crops with high rates of inorganic fertiliser that selects for tall, nitrophilous species that primarily compete with the crop for space and light^21^. To account for the wide range of variation in weed species many of the parameters in the weeds model are drawn stochastically from distributions^20^. Many agronomic decisions can be incorporated into the model, making it a useful tool to examine the effect of changes in agronomy on various metrics of crop production, arable plant biodiversity, and environmental impact. The model runs on a daily time step, takes daily weather variables as inputs, and operates at the scale of a single management unit (a field) with output metrics expressed on a per-hectare basis.

**Figure 1:**
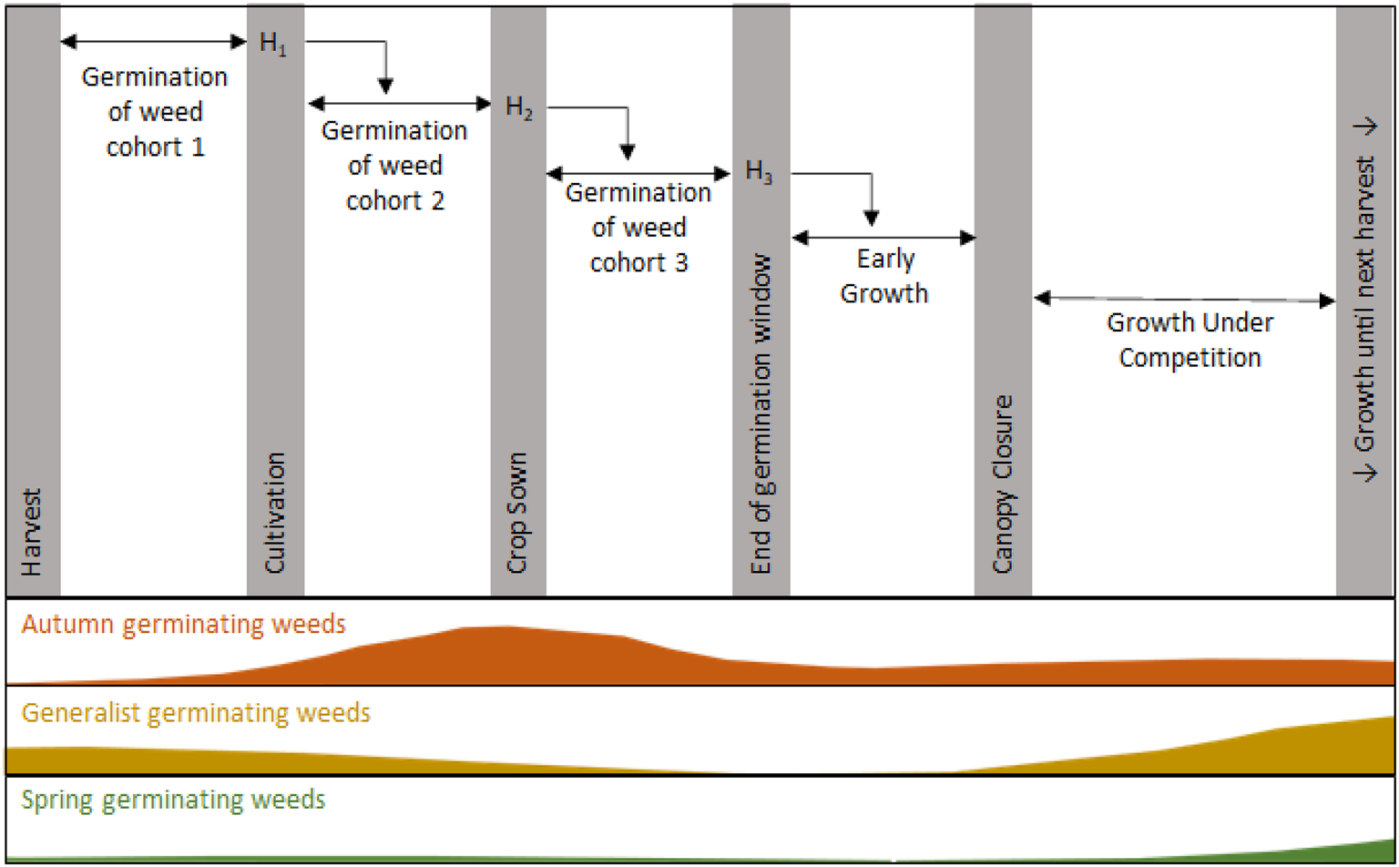
An example of the early part of a winter crop cycle showing three different cohorts of weeds, each emerging in the window between various agronomic operations (grey). Each cohort is recruited from three germination calendars: autumn germinating weeds (orange), generalist germinating weeds (yellow) and spring germinating weeds (green). The composition of each cohort varies according to the timing of management operations. Herbicide applications (H_1-3_) and cultivation cause weed mortality with survivors of these management interventions being recruited into the next weed cohort.

In addition to its basic functionality, we developed the model to include the control of non-crop plants by herbicides using dose–response curves to determine the mortality of each species at a given dose of each herbicide (see Supplementary Methods 1). Herbicides can be applied at each of three time points in the model (Fig. 1): pre-sowing (H_1_), pre-emergence (H_2_) or post-emergence (H_3_). Herbicides applied pre-sowing will act only on seedlings that emerge between the harvest of the previous crop and the pre-sowing cultivation event (weed cohort 1). Any seedlings that survive the H_1_ application will join the next cohort of seedlings which emerge between cultivation and sowing. The H_2_ herbicide application then acts on this cohort of seedlings together with the survivors of H_1_. The survivors of H_2_ then join with the final cohort of seedlings which emerge between sowing and the end of the germination window. H_3_ herbicide applications are applied to this final cohort and any survivors of the previous two herbicide applications. In practice, post-emergence herbicide applications are applied in either the autumn or the spring and this impacts the efficacy, so different dose–response curves are used according to the timing of application (see Supplementary Methods 1). In our simulations, glyphosate is the only herbicide applied in the H_1_ window and it is used as part of the stale seedbed preparation, removing any weeds which emerge before the sowing of the crop. Adjustment to the timing of agronomic operations will alter the size of each cohort of weeds and therefore the number of weeds that survive each herbicide application. Additional herbicide applications at the end of the growing season (for use as a desiccant or to terminate a crop) were not simulated.

### Scenarios

We simulated a winter wheat focussed arable farming system. We matched site conditions to an area of intense agricultural production in the east of the UK with medium clay content soils. We simulated realistic cropping sequences for that region (defined as UKH level 1 NUTS^22^) using a transition matrix describing the probability of growing a series of crops given the previous crop^23^. Our simulation was, therefore, relevant to the large central and south-eastern wheat growing areas in the UK that also experience high weed pressure, including from herbicide-resistant *Alopecurus myosuroides*^24^. We removed the potato crop from the matrix for our simulations as no herbicide efficacy data were available for this crop and it is not a common crop in this region. For each crop, we configured RLM to reflect typical agronomic practices for the region. This included the sowing date, crop cultivar, and sowing rate. We also assumed the use of a typical herbicide program for each crop based on the UK’s Pesticide Usage Survey^4^ and expert opinion (see Supplementary Methods 2). In our simulations we implemented minimum tillage cultivation as standard, whereby the ground is only lightly disturbed before sowing the crop. While ploughing is still practiced in the cropping systems simulated in our study, there is a movement towards reduced tillage systems to reduce emissions and improve soil health. These reduced-tillage systems are currently very reliant on glyphosate for weed control and we wanted to explore the opportunities for retaining their benefits for soil health in the context of the removal of glyphosate.

### Glyphosate scenario (G)

For our baseline scenario, G, we included all standard agronomic practices, as described above, including a typical herbicide program with the use of glyphosate as a pre-sowing weed control.

### No glyphosate scenario (NG)

To explore the impact of removing glyphosate without replacement, we defined the 'No Glyphosate' (NG) scenario. The environment, crop sequences, and management practices mirrored the G scenario in all aspects, including the use of a typical herbicide program only with glyphosate use completely removed.

### Enhanced Integrated Weed Management scenarios (RG/RS/SD/SP)

Integrated Weed Management combines chemical and cultural control methods. An enhanced IWM system (IWM+) aims to target weeds at several stages of their life-cycle and considers multiple weed control tactics^2^.

To investigate alternative weed control strategies without glyphosate, we implemented four IWM+ scenarios based on the principles of crop rotation (R) and stale seedbed management (S). We chose two management practices related to each of these principles, giving 4 IWM+ scenarios (RG, RS, SD, SP in Table 1). In these scenarios, we retained all other typical agronomic practices and herbicides as described above.

**Table 1:** Scenarios included in our simulations including key Integrated Weed Management (IWM) principles that could be adopted following a withdrawal of the use of Glyphosate in the UK.

Scenario	Glyphosate use	Enhanced Integrated Weed Management	The expected effect on the weed community
G	Yes	No	Good weed control
NG	No	No	Poor weed control
RG	No	Yes Crop rotation changed to increase the frequency of grass leys by increasing the probability of transitioning into a grass ley from any other crop by 100%	Promotes the decline of the weed seedbank over time due to cutting the grass, including weeds, before they can produce seeds
RS	No	Yes Crop rotation changed to increase the frequency of spring cereals by increasing the probability of transitioning into a spring cereal from any other crop by 100%	Extends the germination window before sowing, enabling better control of emerging seedlings through H_1_ herbicide applications
SD	No	Yes Stale seedbed management by delaying drilling of winter wheat crops by 3 weeks	Yields similar outcomes to RS but allows for the maintenance of higher profit margins associated with winter crops
SP	No	Yes Stale seedbed management by switching from minimum tillage to ploughing	Inversion tillage mechanically controls weeds before sowing, eliminating seedlings emerging in cohort 1

### Weed community

We initialised the weed seed bank with a total weed seed abundance of 50 000 seeds (typical seed bank densities range from 500 to 500,000^25^). The relative species distribution of those seeds was allocated to reflect seedbank data, collected from over 250 farms in the UK^26^, from sites in the simulated locale (NUTS region H, medium clay soils). *Poa annua* dominated this weed community*.* In recent years, widespread levels of herbicide resistance to several modes of action in *Alopecurus myosuroides* have allowed it to become dominant in many farms^27^. To reflect this, and to investigate whether herbicide resistance status would impact the effect of glyphosate-free strategies, we also repeated the full set of simulations with a starting seed bank more reflective of those sites dominated by *A. myosuroides*. To achieve this we used the same initial seed bank, only with the seed density of *A. myosuroides* substituted for that of *P. annua* in our first set of simulations. Considering the widespread resistance to herbicides in UK *A. myosuroides* populations, we included resistant biotypes in our study, initialising 79% of the *A. myosuroides* population to have herbicide resistance^27^. From hereon we will refer to the simulations using the susceptible *P. annua-*dominated weed community as “Farm S” and the simulations using the herbicide-resistant *A. myosuroides-*dominated weed community as “Farm R.”

### Simulations

For each scenario (G, NG, RG, RS, SD, SP) on each farm (S, R), we ran 100 simulations. We simulated 11 years (10 full cropping seasons) in each simulation to study the immediate consequences to a grower of changing crop management. Whilst longer simulations may have reduced the variation in the observed output, management decisions are usually made with a short–medium term outlook and, as such, any stochasticity in our results will reflect the reality that farmers are continually adapting their systems. The first crop in each simulation was winter wheat to initialise the models at a common starting point with the following crops chosen using the crop rotations generator as described in the scenarios above. We used weather data from Rothamsted weather station (Harpenden, Herts, UK) which records weather data at daily intervals. For the first 20 simulations within each scenario, weather data starting in 1970 were used. Subsequent sets of simulations were done using weather data starting in 1977, 1984, 1991 and 1998 for each set of 20 simulations. We chose this offset of 7 years to make the best use of the period for which weather data were available (1970–2010) whilst minimising overlap with our chosen timescales for the output metrics described below.

Across the scenarios (G, NG, RG, RS, SD, SP), we grouped realisations to allow for direct comparison between the scenarios, i.e. scenario realisations that share an ID were initialised using the same seed. For example, simulation 1 in scenario G (Farms S and R) and simulation 1 in scenario NG and all IWM scenarios (Farms A and B) are directly comparable – they have the same crop sequence, and all stochastic elements were the same, except for the presence or absence of glyphosate. In the RG and RS scenarios, the crop sequences can diverge from other realisations with the same ID due to the use of a different probability matrix in crop selection, but all other stochastic elements remain the same within paired simulations. This allows us to investigate the effect of the change in management in isolation from all the stochastic elements of the model as well as effects due to environmental variation (weather), whilst still examining the effect of these in the different simulations.

### Model output

The RLM, with the additional weed community dynamics model, produces several output metrics relating to both the productivity and environmental impact of the simulated farming system. We chose to sample each simulation at the time of harvest in years 3, 5, and 10 of the simulation. These periods represent short-, medium-, and long-term outlooks: 3 years is typically the shortest repetition of a crop sequence that an arable farmer might follow, 5 years represents the average agreement for farm business tenancies (Defra, 2018; 54% of agreements within the sample had a recorded length of term), 10 years represents the average length tenancy of better-equipped holdings (with a house and buildings; CAAV, 2019). At each sampling time point we took a snapshot of the emerged weed community (abundance in plants ha^−1^ and species richness) and calculated the average yearly food production (calories ha^−1^), profit (£ ha^−1^), and herbicide risk to the environment (Environmental Impact Quotient; EIQ) applied up to the time of sampling.

To convert crop yields into calories ha^−1^, providing an estimate of the total food produced irrespective of the crops grown, we considered waste at the farm gate, extraction rates, wastage in preparation, and the varying calorific content of different crops following^28^.

To calculate the profits (£ ha^−1^) earned in each simulation we used values from^29^ for prices (£ t^−1^) and variable costs (£ ha^−1^) associated with each crop. Under the different scenarios, we adjusted the variable costs as follows: Glyphosate costs were deducted from the variable costs in the glyphosate-free scenarios (NG, RG, RS, SD, SP) at a rate of £2.03 per litre. We assume that machinery costs have already been accounted for based on the baseline cultivation type. However, in scenario SP, which requires additional machinery, we included additional contractor costs^29^ at a rate of £11.33 per hectare (the difference between ploughing, at £50.42 ha^−1^, and seedbed preparation contractor costs, at £39.09 ha^−1^).

For all calculations of calories produced and profits, we assumed the varieties chosen were for human consumption and not feed varieties.

To calculate the herbicide risk to the environment at the level of the active ingredient we adapted methods introduced by Kovach^30^ (see Supplementary Methods 3). These so-called Environmental Impact Quotients (EIQs) provide a measure of the impact of agrochemicals on five targets (groundwater, fish, birds, bees, and arthropods). Unlike other simple pesticide-risk indicators, the EIQ metric is based on both ecotoxicology testing and the likelihood of the chemical reaching each target. In a comparison of various pesticide risk indicators^31^ the EIQ was recommended for use in studies such as ours for its simplicity and consistency. We summed EIQs for overall herbicides applied in each growing season to provide an overall EIQ for each target within each growing season, as well as a comprehensive EIQ score for the growing season by summing the five individual scores.

### Analysis

We performed the analyses of our outputs using the R software^32^. To investigate the effect of the various management options and the starting weed population, and how these might vary over time from implementation we fitted linear mixed models (LMM) to our data on weed abundance, arable plant diversity, food production, farm profits, and EIQ of herbicides. We considered Scenario (G, NG, RG, RS, SD, SP), Farm (S, R), and Simulation time (3, 5, 10 years) in the fixed effects model as well as the second and third-order interactions between them. To consider the paired nature of our simulations the simulation number nested within the weather dataset was included as a random effect. To satisfy assumptions of normality all response variables were log_10_ transformed. We fitted the models using the {lme4} package and tested the significance of the fixed effects using Satterthwaite’s method in the {lmerTest} package in R^33^. We used the {predictmeans} package^34^ to provide model predictions and LSDs for all significant model terms. We also did pairwise comparisons to assess which levels of each model term were significantly different from one another, using a significance level of P = 0.05 (P values were adjusted for pairwise comparisons using Tukey’s method in the {predictmeans} package).

## Results

We took 3054 samples of each output metric from across all our simulations (the missing data points are accounted for where there was no crop to harvest at the sampling time point, typically in grass leys). These samples primarily came from winter wheat crops which represented 44.5% of all crops in the scenarios where we followed typical crop sequences (scenarios G, NG, SD, and SP). In the RG scenario, where the probability of transitioning from any given crop into the grass was increased by 100%, the frequency of grass crops in our simulations increased to 5.7% compared to 2.7% in the other scenarios. Similarly, in the RS scenario, the frequency of spring cereals (Spring Barley + Spring Wheat) increased to 16.5% compared to 10.6% in the unaltered rotations.

**Figure 2:**
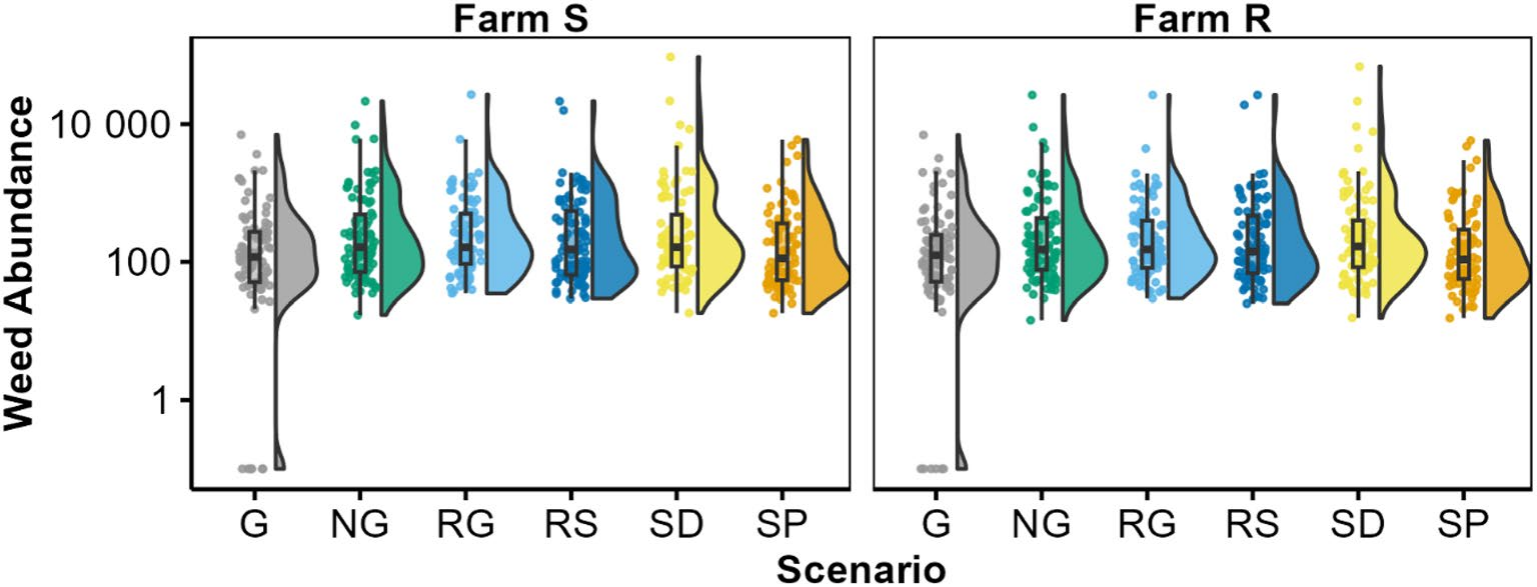
Emerged Weed abundance (ha^−1^) following 5 years of simulation varies by Farm and Scenario. Each data point shows the sampled weed abundance in a single simulation. Box and whisker plots show the median, interquartile range and 95th percentiles of the data. Density plots describe the distribution of data points over the range. Farm S has a weed community dominated by *Poa annua* and Farm R has a weed community dominated by herbicide-resistant *Alopecurus myosuroides*. The different Scenarios are shown in grey = glyphosate (G), green = no glyphosate (NG), blues = changed crop rotation where light blue = increased frequency of grass leys (RG) and dark blue = increased frequency of spring crops (RS), yellows = stale seed bed practices where light yellow = delayed drilling of winter wheat crops by 3 weeks (SD), and dark yellow = switch from minimum tillage to ploughing (SP).

A common result across all the output metrics was a large amount of variation in the outputs, often with considerable variation within each scenario, farm, and timescale (see Fig. 2), largely driven by stochasticity within the model and environmental drivers. By accounting for weather and simulation number in the random effects of the LMMs we could identify differences attributable to the Scenario, Farm, or Timescale that would otherwise be obscured by the large variation (Table 2).

**Table 2:** The significance (P-values, 3dp) of fixed-effect terms from Linear-Mixed Models.

n = 3054	Fixed effect						
Output metric:	Scenario	Farm	Timescale	Scenario × farm	Scenario × timescale	Farm × timescale	Scenario × farm × timescale
Weed abundance	** < 0.001**	0.409	0.297	0.996	0.538	0.655	1.000
Weed species richness	** < 0.001**	0.536	0.593	1.000	0.614	0.999	1.000
Food production	** < 0.001**	** < 0.001**	** < 0.001**	0.991	0.090	0.138	1.000
Profit	** < 0.001**	** < 0.001**	** < 0.001**	0.985	0.343	**0.040**	1.000
EIQ-total	** < 0.001**	1.000	** < 0.001**	1.000	**0.004**	1.000	1.000

Where P < 0.05 values are shown in bold. A separate model was fitted to each output metric and the simulation number nested within the weather set was included as random effects.

The LMMs revealed several significant predictors for our output metrics (Table 2). There were strong significant effects of Scenario for all output metrics. Only food production and profits showed a significant effect of Farm. Finally, food production, profit, and EIQ were significantly affected by Timescale. There was also evidence of an interaction between the effect of Farm x Timescale on profits, and of Scenario and Timescale on EIQ.

**Figure 3:**
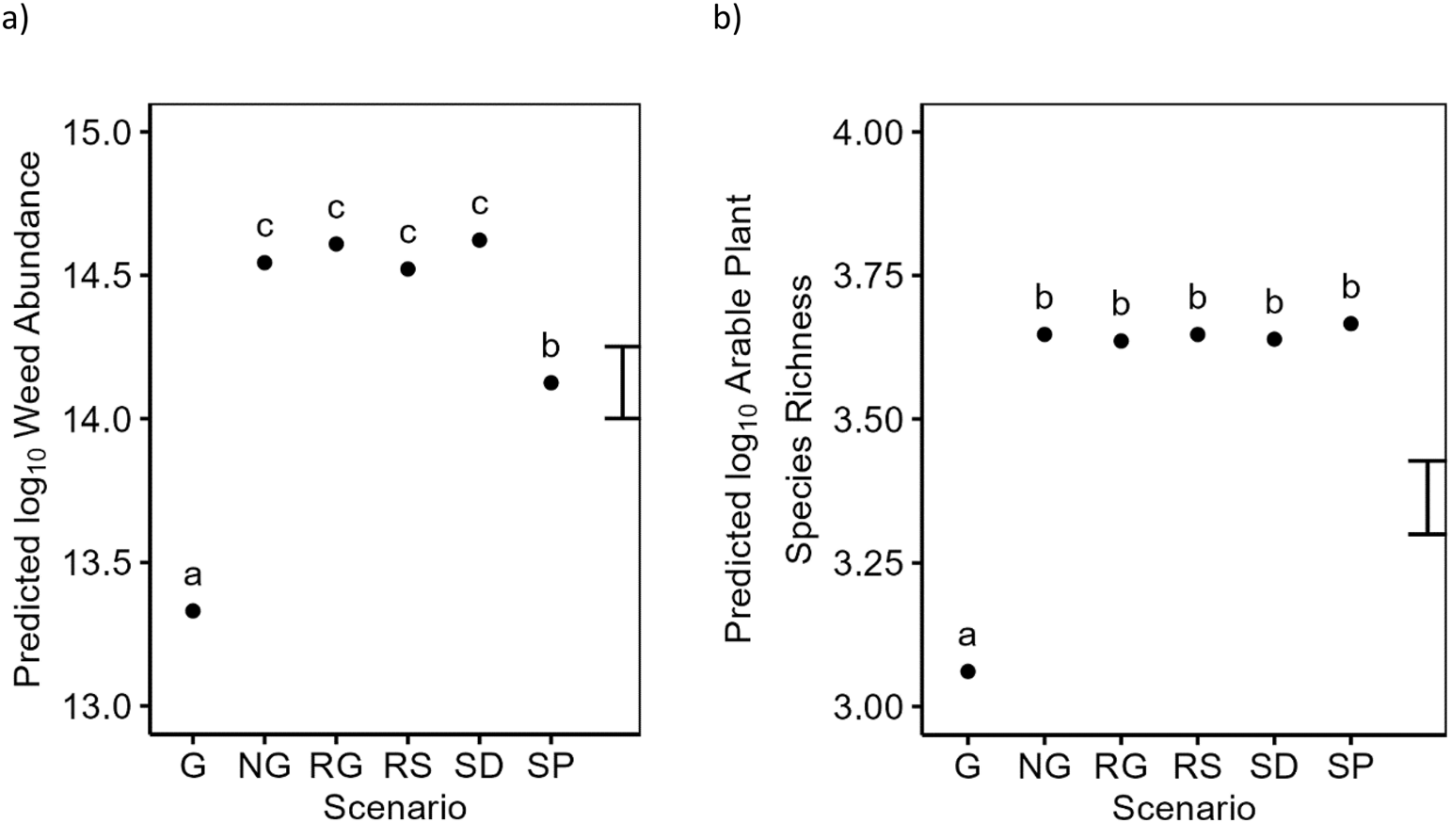
Predicted (**a**) log10 weed abundance (ha^−1^) and (**b**) log10 species richness from a linear model. Model terms are shown in Table 2. Predictions are classified by the main effect of the Scenario. Predictions are averaged over all levels Farm S and Timescale. The error bar shows the approximate average LSD. Means within a single panel labelled with different letters indicates they are significantly different (P < 0.05, post-hoc Tukey test).

In our sampled weed communities we observed differences both in terms of total weed abundance and species richness. Weed abundance varied significantly between Scenarios, with the lowest weed abundances in the presence of glyphosate (G, Fig. 3a). Our only observations of a weed community of close to zero abundance were in the G Scenario (Fig. 2), reflecting the efficacy of glyphosate and lack of any evolved resistance in our simulated weed populations. In Scenario SP, the introduction of ploughing significantly increased weed abundance compared to the G scenario. Nevertheless, the SP Scenario provided the lowest weed abundances of all IWM+ Scenarios (significantly lower compared to SP to RG, RS, and SD in Fig. 3a). Of note is the fact that only scenarios G and SP avoided the risk of high abundances that may represent levels of weed infestation that affect the viability of the crop. Patterns in our observed weed abundances coincided with patterns in weed species richness observations (Fig. 3b). However, when considering weed species richness the SP scenario was not significantly different from the other IWM+ Scenarios. There were no significant differences in either weed abundance or species richness between the two Farms, nor did this change significantly with Timescale.

**Figure 4:**
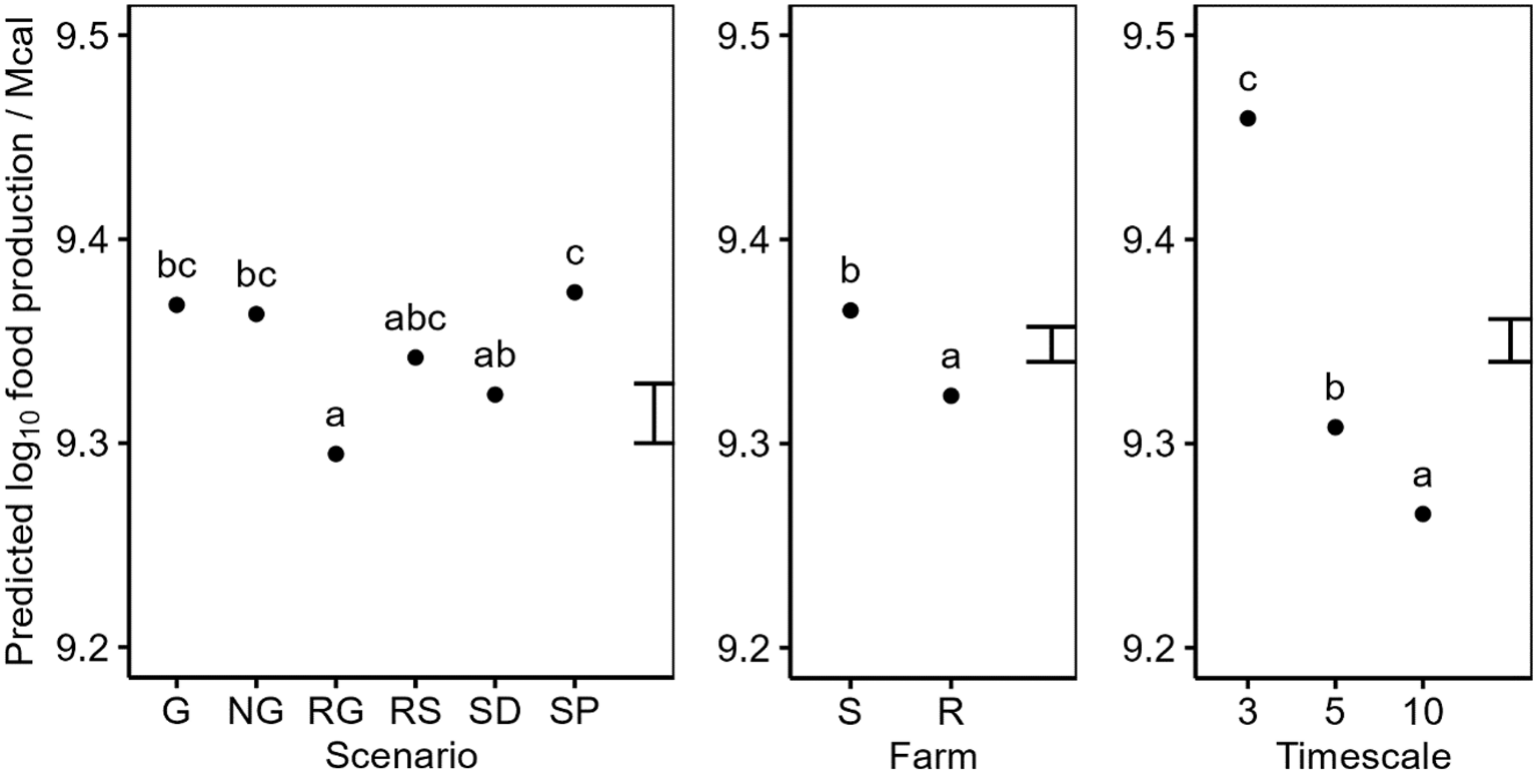
Predicted log10 food production (Mcal ha^−1^ year^−1^) from a linear model. Model terms are shown in Table 2. Predictions are classified by the main effects of the Scenario, Farm, and Timescale. Predictions are averaged over all levels of other terms included in the model. The error bar shows the approximate average LSD. Means within a single panel labelled with different letters indicates they are significantly different (P < 0.05, post-hoc Tukey test).

There was no significant difference between food production in the G and NG scenarios. However, we did observe significant differences between the yields of several crops in the G and NG scenarios (see supplementary results 6). These included beans, spring barley and spring wheat. We observed significant reductions in food production in the grass ley, and delayed drilling Scenarios (compare RG and SD with G in Fig. 4). However, there are many individual simulations where both the RG and SD scenarios showed increased food production (See Supplementary Results 1). Bean crops yielded consistently poorly in the absence of glyphosate across our simulations, reflecting the fact that it is a relatively poor competitor and the lack of selective herbicides available, whilst a few winter wheat crops did not yield at all in scenario SD where drilling was delayed by 3 weeks. Food production was significantly higher in Farm S than in Farm R (Fig. 4). The average food production per year decreased over Timescale, with the greatest level of food production observed in the first 3 years of the simulation (Fig. 4).

**Figure 5:**
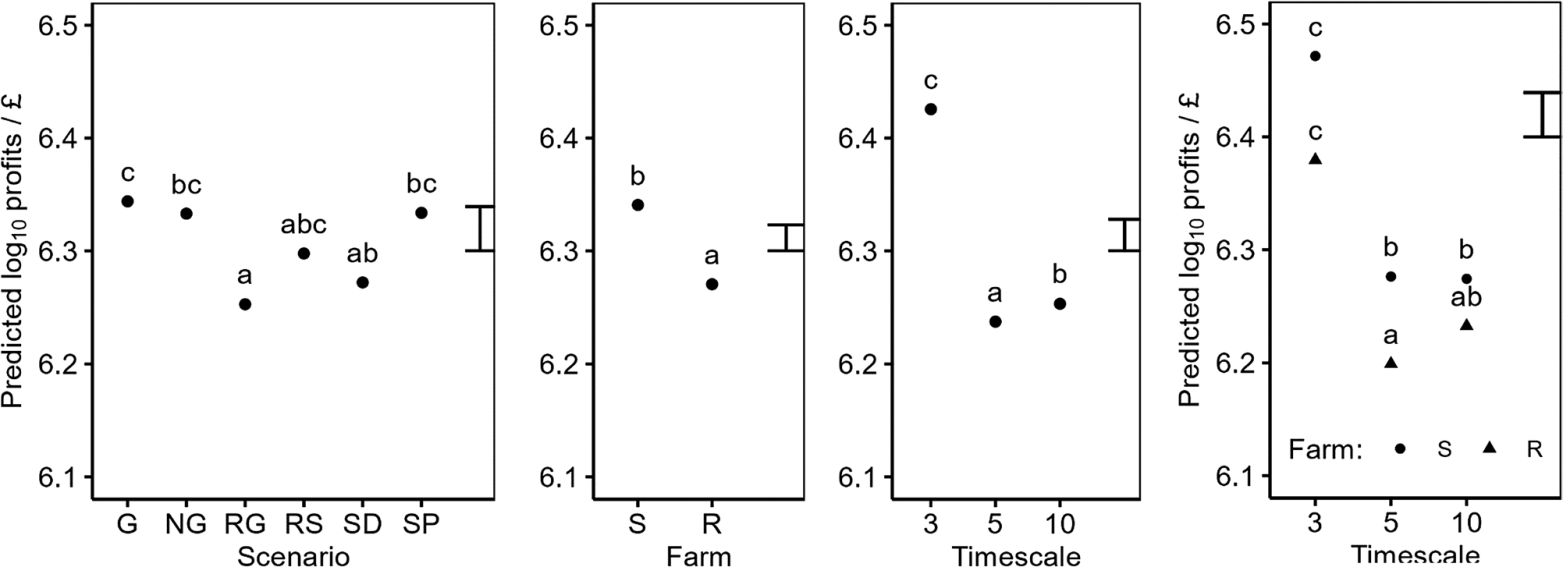
Predicted log10 profits (£ ha^−1^ year^−1^) from a linear model. Model terms are shown in Table 2. Predictions are classified by the main effects of the Scenario, Farm, and Simulation year, and the interaction between the Farm and the Simulation year. Predictions are averaged over all levels of other terms included in the model. The error bar shows the approximate average LSD. Means within a single panel labelled with different letters indicates they are significantly different (P < 0.05, post-hoc Tukey test).

Three of our IWM+ Scenarios (RG, RS, and SD) were significantly less profitable than the Glyphosate-based scenario. Only the IWM+ scenario with the addition of ploughing (SP) was able to provide similar profits to the G scenario (Fig. 5). The observed profits over the three Timescales show a similar trend as for food production. We found the highest profits in the first 3 years of simulation with a drop in profits by year 5, but unlike food production, profits do not continue to fall into year 10. The interaction between Farm and Timescale was significant indicating that the relative profits change over time between Farms S and R—the profit deficit in Farm R shrinks by the tenth year of simulation.

**Figure 6:**
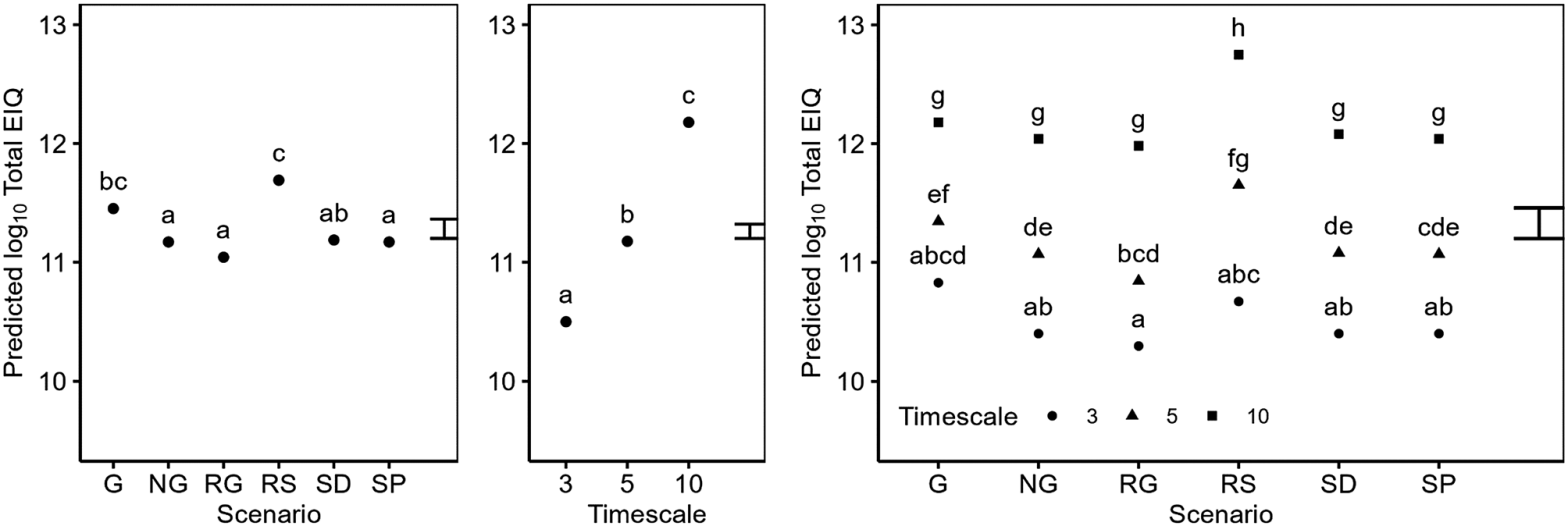
Predicted Total EIQ (Year^−1^) from a linear model. Model terms are shown in Table 2. Predictions are classified by the main effects of the Scenario, and Simulation year, and the interaction between the Scenario and Simulation year. Predictions are averaged over all levels of other terms included in the model. The error bar shows the approximate average LSD. Means within a single panel labelled with different letters indicates they are significantly different (P < 0.05, post-hoc Tukey test).

Average yearly EIQ (Fig. 6) scores varied significantly with both Scenario and Timescale. The G Scenario had a significantly higher EIQ than most other Scenarios (NG, RG, SP) and was only surpassed by the RS Scenario. A significant interaction between the Scenario and Timescale main effects indicates that the relative herbicide risk associated with scenarios changes over time. In the short term (first 3 years of simulation), the G scenario had the highest EIQ score on average (not significant). However, as the simulations progressed the EIQ scores found in the RS scenario increased more rapidly, becoming significantly higher than all other non-glyphosate scenarios (NG, RG, SD, SP) by year 5, and by year 10 of our simulations the RS scenario had a significantly higher EIQ than all other scenarios. This reflected the worse environmental risk profile of post emergence herbicides used in the spring crops.

## Discussion

Glyphosate has been associated with concerns regarding environmental impact^12^, herbicide resistance^14^, and human health^7^ and so there is a desire from policy makers, growers and the public to reduce its use. Additionally, the preservation of arable plant biodiversity and the transition to integrated weed management practices have gained attention due to their potential benefits for ecosystem function and sustainable agriculture^35–37^. With this context in mind, our study investigated the implications of removing glyphosate, from a typical arable non-GM system, on weed abundance, arable plant diversity, food production, profitability, and herbicide risk to the environment.

Our first hypothesis predicted that removing glyphosate would lead to increased weed abundance and arable plant diversity, and a decrease in food production, together with a decrease in the risk associated with herbicides to the environment. Our findings align with this hypothesis and support previous studies that have highlighted the importance of glyphosate in weed control and its indirect impact on crop yield^38,39^. The observed increase in weed abundance in our simulations confirms the effective simulation of high weed mortality rates due to glyphosate^38^. Additionally, the reduction in crop yields and profits emphasizes the potential socioeconomic implications of glyphosate loss, such as a predicted $6.76 billion annual loss of global farm income^40^. Whilst the yields of some crops were reduced in the NG scenario, the overall change in food production was not significant. This finding, together with the significant increase in arable plant diversity in the corresponding simulations supports the role of weed diversity in facilitating crop production^37^. The reduction in EIQ score following glyphosate removal (difference between G and NG Scenario) and increases in arable plant diversity additionally confirm the damaging role glyphosate plays in the farmed environment^41,42^. The occasional high weed abundances in all scenarios lacking glyphosate (with the exception of SP) and examples of total weed control in scenario G, illustrated the current value of glyphosate to farmers to arrest the growth of weed populations in the short term, potentially ‘rescuing’ a field from problematic weed infestations.

Our second hypothesis proposed that the loss of glyphosate would have the greatest impact on farms with high levels of resistance to other herbicides. On these farms, where there is reduced weed control by other active ingredients, there is more reliance on alternative modes of action, including glyphosate, in the weed control program^43^. However, our results did not support this hypothesis. Although we observed significantly reduced food production and profits on the Farm with high herbicide resistance, the lack of interaction between Farm and Scenario suggests that the loss of glyphosate may not necessarily exacerbate this problem. The baseline cropping system studied here is a simple one, which has led to poorly diverse weed communities prone to developing herbicide resistance^27^ and so whether the weeds in question are resistant or not does not underpin the reliance on glyphosate but rather the glyphosate-use masks the yield losses due to a lack of weed diversity across both Farms^37^. Furthermore, there are indications that the production gap between Farms with and without herbicide resistance could potentially decrease in the long term. These findings indicate the complex dynamics of herbicide resistance and the need for further investigation into the interactions between resistance levels and the consequences of glyphosate removal.

In our third hypothesis, we proposed that the impacts of glyphosate loss could be mitigated through the implementation of IWM practices. We investigated the effects of changing crop rotation to include more spring cereals or grass leys, as well as the use of stale seedbed techniques and ploughing. These simulations showed mixed results and so we cannot categorically confirm our third hypothesis but rather support the view that the implementation of some IWM practices may mitigate the impacts of glyphosate loss to some extent. This is in accordance with a previous modelling study by Beckie et al.^44^ who found that it may be possible to maintain profit margins in southern Australia using cultural weed management, albeit with higher weed densities.

The uncertainty associated with the non-chemical options we tested (particularly delayed drilling) supports the view that adoption of IWM, requires a knowledge-intensive approach that integrates multiple options adapted to the local environment^2^. This will however require careful consideration and a strong founding in the principles of weed ecology and biology. Introducing more grass leys (RG) or spring cereals (RS) into the crop rotation inevitably led to a decrease in food production due to the replacement of high-yielding crops, with lower- or non-yielding alternatives. Whilst in some cases the additional benefits of these diversified systems may outweigh the loss of food production, there may also be additional avenues to increase productivity in these systems. For example, the addition of grazing animals to grass leys will not only provide additional revenue sources but may also improve soil structure and nutrient cycling^45^.

We expected to observe weed control benefits of adjusting the crop rotation, yet these did not consistently materialize in our simulations. We anticipated that the introduction of crops with very different management techniques to the typical winter cereals of our chosen system would have allowed weed abundance to fall as the management operations required for these alternative crops will select a different weed community to that which is adapted to winter cereals^46,47^. Despite no significant decrease in weed abundance, the RG and RS scenarios did contribute to increased arable plant diversity. Scenarios RG and RS effectively represent a diversification of the habitat niche for weeds and it has been argued that this should lead to greater weed functional diversity and lower competitive yield losses in any given crop^37,48^—a hypothesis partly supported by our results. It should be noted that simulations in the spring cropping RS Scenario resulted in an increase in EIQ. The typical herbicide program applied here, and currently applied by UK farmers, in spring crops contains active ingredients with remarkably high EIQ scores due to their high toxicity and bioavailability. Thus, there appear to be significant ecological implications of using this approach. However, the benefits of spring crops (and other IWM techniques) in improving the arable plant diversity are clear from our results. It is also well-documented that the overwinter stubbles (made possible by spring cropping) provide conditions for the germination of crucial winter food sources for seed-eating birds and that the spring crops (including spring emerging weeds) themselves can provide preferred breeding and foraging habitats for farmland birds^49,50^. It is therefore important to consider these trade-offs when implementing such a strategy on farm. The application of alternative chemical control programs with lower EIQs also warrants further investigation.

In our simulations, delaying drilling of winter wheat crops by 3 weeks (SD) led to increased weed abundance and a decrease in food production (compared to G scenario). This IWM practice relies on the stale seedbed concept, where the window for germination prior to drilling is extended. These weeds are then removed before the crop emerges. The current implementation of this management practice relies heavily on the use of glyphosate prior to drilling (H_1_ in Fig. 1) and so it follows that the use of this technique in a glyphosate free system does not confer any advantages in terms of weed control. It is worth noting that we did see increased arable plant diversity in this scenario, and that is likely due to a change in the germination window allowing weeds with alternative germination calendars to be recruited into the first cohort (Fig. 1).

The other IWM practice that focuses on the principle of a stale seedbed (SP) replaced the pre-drilling glyphosate application (at H_1_ in Fig. 1) with the mechanical weed control provided by the plough during tillage^51^. This had positive impacts on reducing weed abundance and correspondingly increased food production. However, the benefits associated with ploughing rarely translated into increased farm profits due to the associated higher costs. Despite the SP scenario showing many key benefits, notably the largest reduction in weed abundance in the absence of glyphosate, there are likely to be many reservations preventing farmers from adopting this technique, with the impacts on soil health being a primary concern^52,53^.

By using paired simulations, we were able to disentangle some consistent responses in outputs to each scenario. However, despite these significant results, we cannot ignore the huge variation in response between simulations. Our simulations indicate that stochastic elements and changing weather conditions are major sources of variability in agricultural systems. For each output metric, we observe simulations that outperform the glyphosate scenario and some that perform worse. This echoes experimental studies which have looked at the utility of various IWM practices and have often find contradicting results. For example, Lutman et al.^54^ found that ploughing had been reported as causing *A. myosuroides* populations to increase by up to 82% in one study or decrease by as much as 96% in another—an artefact of different starting points in terms of density and distribution of weed seed in the soil.

In this study, we chose to use simple metrics to represent key levers in an agricultural system: weed abundance and species richness to represent weed communities, food production and profits to represent productivity, and EIQ to represent herbicide risk and environmental impacts. Each of these metrics represents an oversimplification of what is an extremely complex and multifaceted system. More complex methodologies are available allowing in-depth and nuanced examination of the impacts of removing glyphosate that cannot be achieved in a multi-dimensional study such as the one presented here. For example a combination of phylogenetic and trait based approaches has been demonstrated to provide insights into weed community assembly^55^, and economic modelling can be used to explore cost effectiveness and the feasibility of various weed control options^56^. One area in particular which would benefit from in-depth exploration in the future is the environmental impacts of glyphosate removal. Here, we used the EIQ score for its simplicity and ease of interpretation, yet criticisms of this metric are common in the literature^57,58^ due to the failure of EIQ to capture the complexities of environmental fate and other aspects of environmental impact beside ecotoxicology. More complex Life Cycle Analysis (LCA) tools are readily available and have been used to explore the impacts of novel crop introductions^59^, organic and low-input production^60^ and integrated weed management^61^. The application of LCA to our study system would allow a much broader exploration of the environmental benefits of glyphosate withdrawal.

Our study indicates that, whilst food production and profits are significantly reduced under a glyphosate-free scenario there are indeed several positive outcomes in terms of reducing herbicide risk to the environment and increasing arable plant diversity. Whether glyphosate will be withdrawn or through a will to change the status quo, farmers are beginning to investigate alternative weed management strategies. We have identified that it is imperative to carefully assess these management changes through simulations to understand trends, as it is not always possible to predict singular outcomes when stochastic elements and weather are playing a key role. Whilst data from pot and plot trials are extremely valuable for understanding the underlying principles, they may provide misleading results in terms of system outputs, and it is only through paired analysis that patterns begin to emerge.

## Conclusion

Our study suggests that removing glyphosate from arable systems has significant implications. While it results in increased weed abundance, reduced crop yields and lowered profits, it can also offer positive outcomes, such as reduced herbicide risk to the environment and increased arable plant diversity. Farmers who are considering alternative weed management strategies should carefully assess these options, considering both ecological and economic factors. The integration of simulation studies, empirical research, and a thorough understanding of weed ecology and biology will contribute to informed decision-making and sustainable agricultural practices.

### Supplementary Information

Supplementary Information.

## Data Availability

The datasets used and/or analysed during the current study available from the corresponding author on reasonable request.
