# Supplementary material for: Trade-offs constrain the success of glyphosate-free farming
Source: Sci Rep. 2024 Apr 5;14:8001. doi: 10.1038/s41598-024-58183-8 (PMC10997608; doi:10.1038/s41598-024-58183-8)
Supplement: Supplementary file 1 — Supplementary Information. [file 41598_2024_58183_MOESM1_ESM.pdf]

Supplementary Materials

Supplementary Methods 1: Dose Response Curves

For each herbicide applied in our model we downloaded dose response data for all species from the crop protection online portal (https://plantevaernonline.dlbr.dk/). These data are at the level of herbicide products rather than active ingredients and so we determined which products would give the correct field use rating as determined by Ritz et al 2015 in Supplementary Methods 2. These data are specific not only to the herbicide product but also to the timing of the application and the crop in which the herbicide is applied. We assigned each of these herbicide applications into one of 4 windows: pre-sowing, pre-emergence, post-emergence (autumn) and post-emergence (spring). We did not include any herbicides used as a pre-harvest desiccant. For post-emergence herbicides, the crop growth stage was assumed to be 13 if the herbicide was applied in the autumn (for winter crops) otherwise, for post-emergence herbicides applied in the spring the crop growth stage was assumed to be 29. For post-emergence herbicide applied in sugar beet, the growth stage was assumed to be 15. The downloaded data provide percentage kills for quarter, half, full, and double rate applications (of the recommended field rate application).

We took these dose-response data for all species of weed simulated in our model and fitted a dose-response curve using the {drc} package (Ritz et al 2015). For a given herbicide we chose the best fitting model from the following options: 3- 4- or 5-parameter log-linear, 4-parameter Cedergreen-Ritz-Streibig, 2- 3- or 4-parameter Weibull functions and allowed parameters to vary for each weed species. For species where data were not available, we took the average value for each parameter (b, d, and e) across all weed species of the same phylogenetic type (grass/broadleaf). Where there were no species of the same type, we assumed that the product in question was not effective against weeds of that type and so no individuals of that type are killed by that product in our model.

No data were available for herbicides applied outside of the cropping period (pre-sowing herbicide application window). In our standard herbicide programs, the only herbicide applied in this window was glyphosate applied as part of a “stale seedbed” management practice. As there are no known cases of herbicide resistance and susceptibility to this active ingredient in the UK remains high, we assumed 100% control.

Ritz, C., Baty, F., Streibig, J. C., Gerhard, D. (2015) Dose-Response Analysis Using R PLOS ONE, 10(12), e0146021

Supplementary Methods 2: Standard Herbicide Programs.

We determined standard herbicide regimes (typical applications for the UK) for each crop in the RLM (Table 1). These regimes consist of the most likely combination of products used based on the data recorded by Defra in their pesticide usage survey (see Table 1 for references). For each crop we have looked at the average number of herbicides applied and combined this information with the most commonly applied products to deduce a likely regime. For many of the crops there were a range of similar products applied in similar quantities on average and so we chose only one of those and made sure to cover all the modes of action commonly included.

For each active ingredient (there may be multiple active ingredients within one herbicide product) we then calculated a field use rating (F, g ha^{-1}):

$$f = n \times p \times r \times h \quad (1)$$

where: n, is the number of times the product is applied in the growing season; p, is the proportion of the recommended field rate that is applied in each application; r, is the recommended field rate (l ha^{-1}) for the product; and, h, is the amount of active ingredient present in the product (g l^{-1}) and present in the product.

Table 1: Standard pesticide programs derived for crops included in RLM. Grass crops as part of an arable rotation were determined to not receive any pesticides (Grassland and fodder crops in the UK 2017).

**Crop**	**Reference**	**Application window**	**a.i.**	*f*
Winter Wheat	Arable crops in the UK 2018	pre-sowing	glyphosate	1080
		pre-emergence	diflufenican	60
		post-emergence Autumn	mesosulfuron-methyl	12
			iodosulfuron-methyl-sodium	2.4
		post-emergence Spring	florasulam	4.5
			fluroxypyr	180
Winter barley	Arable crops in the UK 2018	pre-sowing	glyphosate	1080
		pre-emergence	diflufenican	60
		post-emergence Spring	florasulam	4.5
			fluroxypyr	180
Oilseed rape	Arable crops in the UK 2018	pre-emergence	clomazone	118.8
		post-emergence Autumn	propaquizafop	150
			propyzamide	850
Beans	Outdoor vegetable crops in the UK 2017	pre-sowing	glyphosate	270
		pre-emergence	clomazone	118.8
Spring barley	Arable crops in the UK 2018	pre-sowing	glyphosate	1080
		Post-emergence Spring	metsulfuron-methyl	4.76
			thifensulfuron-methyl	47.74
Spring wheat	Arable crops in the UK 2018	pre-sowing	glyphosate	1080
		post-emergence Spring	metsulfuron-methyl	4.76
			thifensulfuron-methyl	47.74
Sugar Beet	Arable crops in the UK 2018	pre-sowing	glyphosate	270
		post-emergence (Spring)	metamitron	1400
			phenmedipham	135

			desmedipham	135
			ethofumesate	168.75
maize	Grassland and fodder crops in the UK 2017	pre-sowing	Glyphosate	270
		post-emergence (Spring)	Mesotrione	78.75

Supplementary Methods 3: Environmental Impact Quotients

For each active ingredient we can calculate environmental impact scores to represent the environmental impact of that active ingredient. We wanted to be able to measure the impact of each pesticide program on various environmental properties. We followed the methods established by Kovach et al (1992) to calculate environmental impact quotients (EIQ) for groundwater, fish, birds, bees, and beneficial arthropods. As we were looking at typical products and therefore active ingredients in use in the UK, not all products were available within the EIQ database provided by the authors which was built in the US and so we calculated these scores using data from the 'Pesticide properties database' (Lewis et al., 2016) as follows:

The groundwater score (EIQ_G) is given according to the leaching potential (L) of the active ingredient. L takes a value of either 1, 3 or 5 depending on the GUS leaching potential index of the active ingredient (<0.1=1, 0-3=3, >3=5).

The fish score (EIQ_F) is given by the product of the surface loss potential (R) and the fish toxicity (F) of the active ingredient. R takes values of 1, 3 or 5 depending on the solubility of the active ingredient in water at 20°C (mg l^{-1}) (<1=1, 1-1000=3, >1000=5) and F takes values of 1, 2 or 3 according to the fish acute 96 hour LC50 (mg l^{-1}) of the active ingredient (>10=1, 1-10=2, <1=3).

The bird score (EIQ_D) is calculated by $EIQ_B = \mathrm{D} \times \frac{\mathrm{S+P}}{2} \times 3$ where D is the bird toxicity, S is the soil half-life and P is the plant surface half-life. We assigned values of D according to the birds acute LD50 (mg kg^{-1}) (>1000=1, 100-1000=3, 1-100=5), S scores of 1, 3 or 5 were assigned according to the Soil degradation (days) (aerobic) DT50 (typical) (<30=1, 30-100=3, >100=5) and P scores were assigned according to the dissipation rate RL50 on and in plant matrix (<14=1, 14-28=3, >28=5) of the active ingredient.

The bee score (EIQ_Z) is given by $EIQ_Z = Z \times P \times 3$ where Z is the bee toxicity which is given a score of 1, 3 or 5 according to the value of the honeybees (Apis spp.) oral acute LD50 (worst case from 24, 48 and 72 hour values - µg bee^{-1}) (>100=1, 1-100=3, <1=5).

Finally, the beneficial arthropod score (EIQ_B) is given by $EIQ_Z = B \times P \times 5$ where B is the arthropod toxicity which is assigned a score of 1, 3 or 5 according to the arthropod LR50 g ha^{-1} (>1000=1, 100-1000=3, 1-100=5).

We also computed a comprehensive EIQ score (EIQ_C) by summing across the five individual EIQ scores: $EIQ_C = EIQ_G + EIQ_F + EIQ_D + EIQ_Z + EIQ_B$.

Where any data were missing, the middle score value was assigned to that metric.

For each product used in the standard pesticide programs we calculate the typical field use rating for each active ingredient by multiplying the typical number of applications by the recommended rate of application for the product (l/Ha) by the dose of the active ingredient within the product (g a.i. /l).

We can multiply the field use rating for each active ingredient by each EIQ score (G, F, D, Z, and B) in turn to give the typical environmental impact on each type. We can sum across all active ingredients applied in a crop to give the typical environmental impact of pesticide use in that crop (total EIQ).

The EIQ scores for each of our standard pesticide programs in each crop are shown in Figure 1.

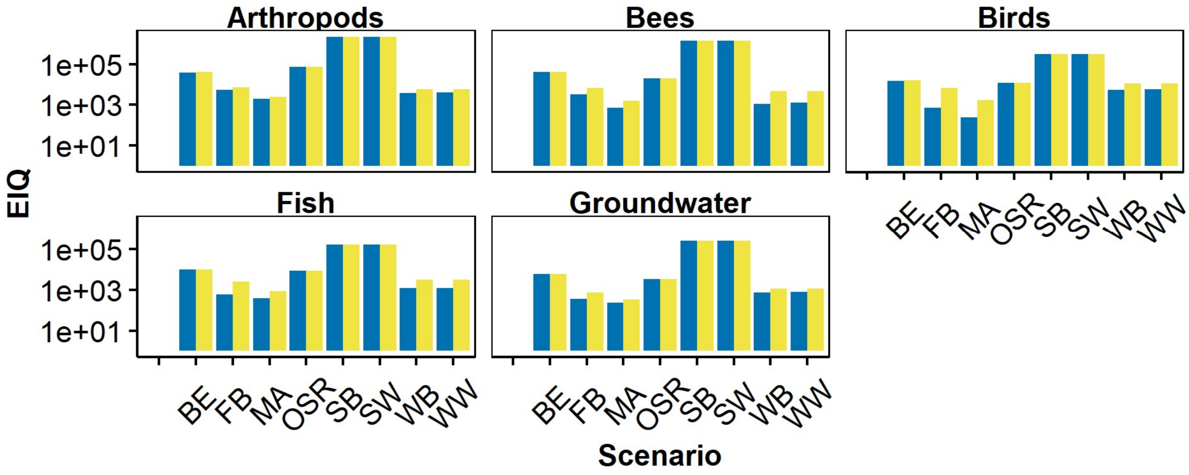

Figure 1: The EIQ scores for arthropods, bees, birds, fish and groundwater for the standard pesticide program determined for each crop, without (blue) and with (yellow) glyphosate. BE= sugarbeet, FB=fieldbeans, MA=maize, OSR=oilseed rape, SB=spring barley, SW=spring wheat, WB=winter barley, WW=winter wheat.

Supplementary Results 1: Weed Abundance

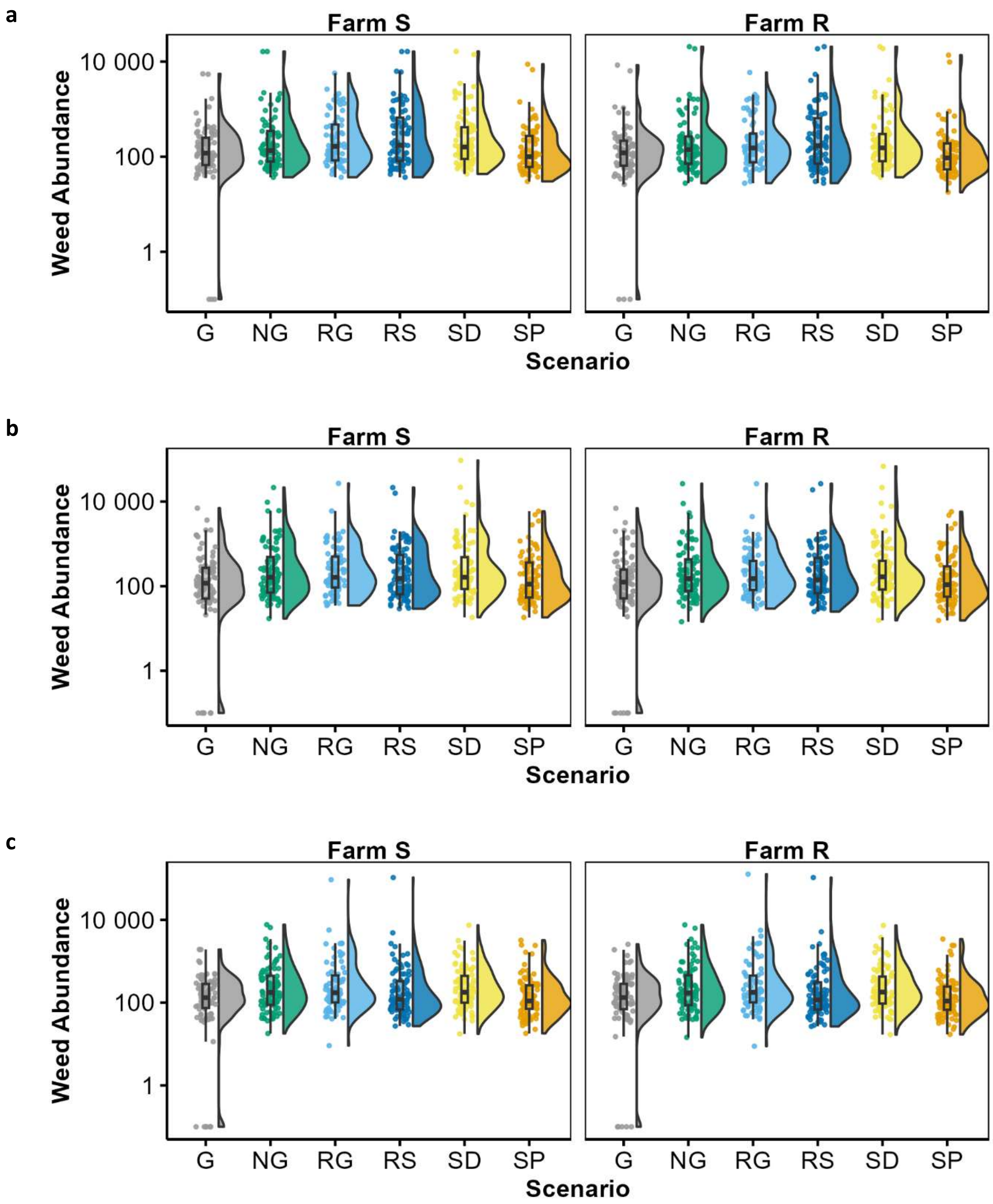

Figure 2: Weed Abundance in each scenario after (a) 3, (b) 5, and (c) 10 years of simulation. Farm S has a weed community dominated by Poa annua and Farm R has a weed community dominated by Alopecurus myosuroides.

Scenarios are: glyphosate (G), no glyphosate (NG), increased frequency of grass leys (RG), increased frequency of spring crops (RS), delayed drilling of winter wheat crops by 3 weeks (SD) and, switch from minimum tillage to ploughing (SP).

Supplementary Results 2: Weed Species Richness

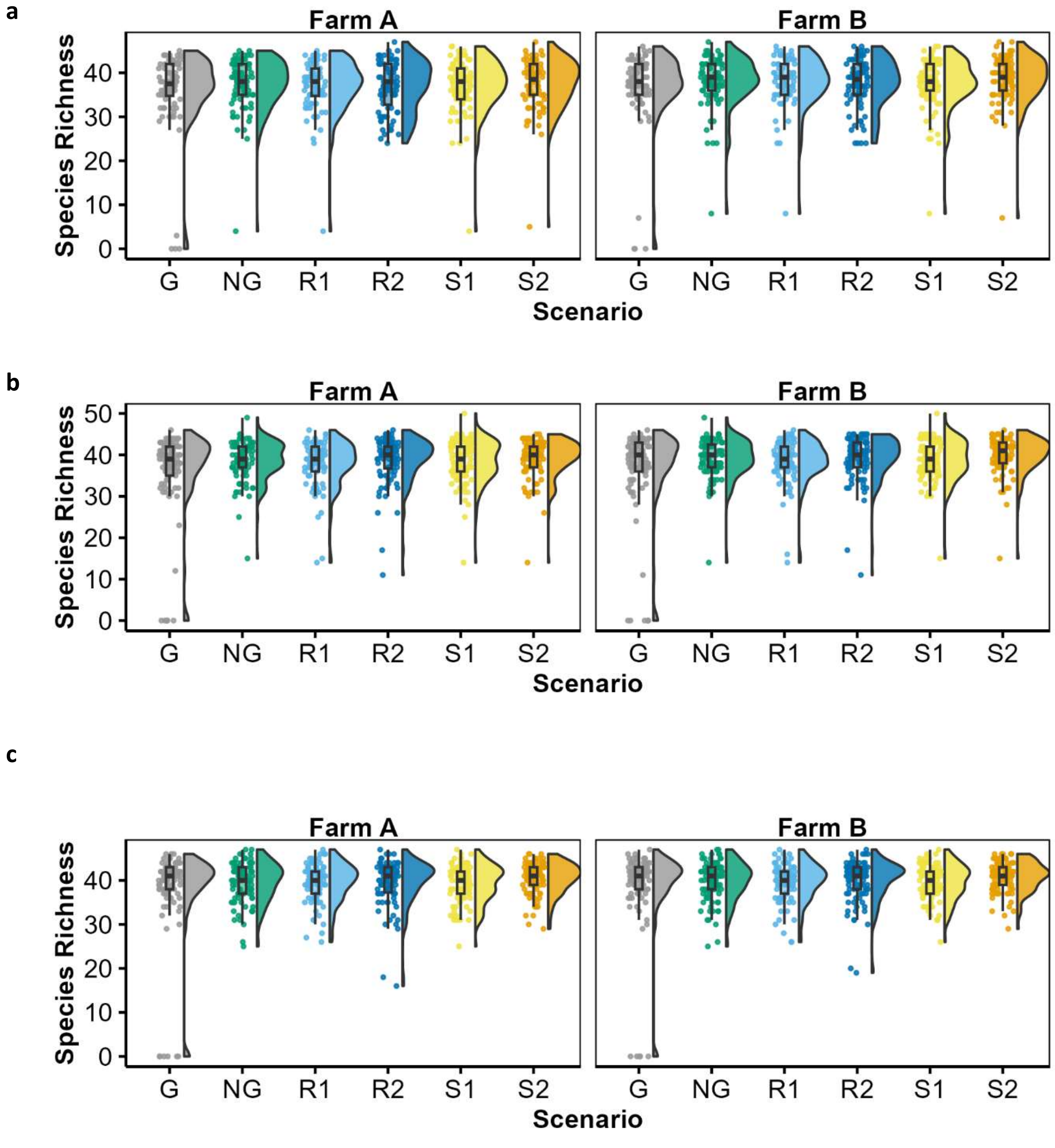

Figure 3: Weed Species richness in each scenario after (a) 3, (b) 5, and (c) 10 years of simulation. Farm S has a weed community dominated by Poa annua and Farm R has a weed community dominated by Alopecurus myosuroides. Scenarios are: glyphosate (G), no glyphosate (NG), increased frequency of grass leys (RG), increased frequency of

spring crops (RS), delayed drilling of winter wheat crops by 3 weeks (SD) and, switch from minimum tillage to ploughing (SP).

Supplementary Results 3: Food Production

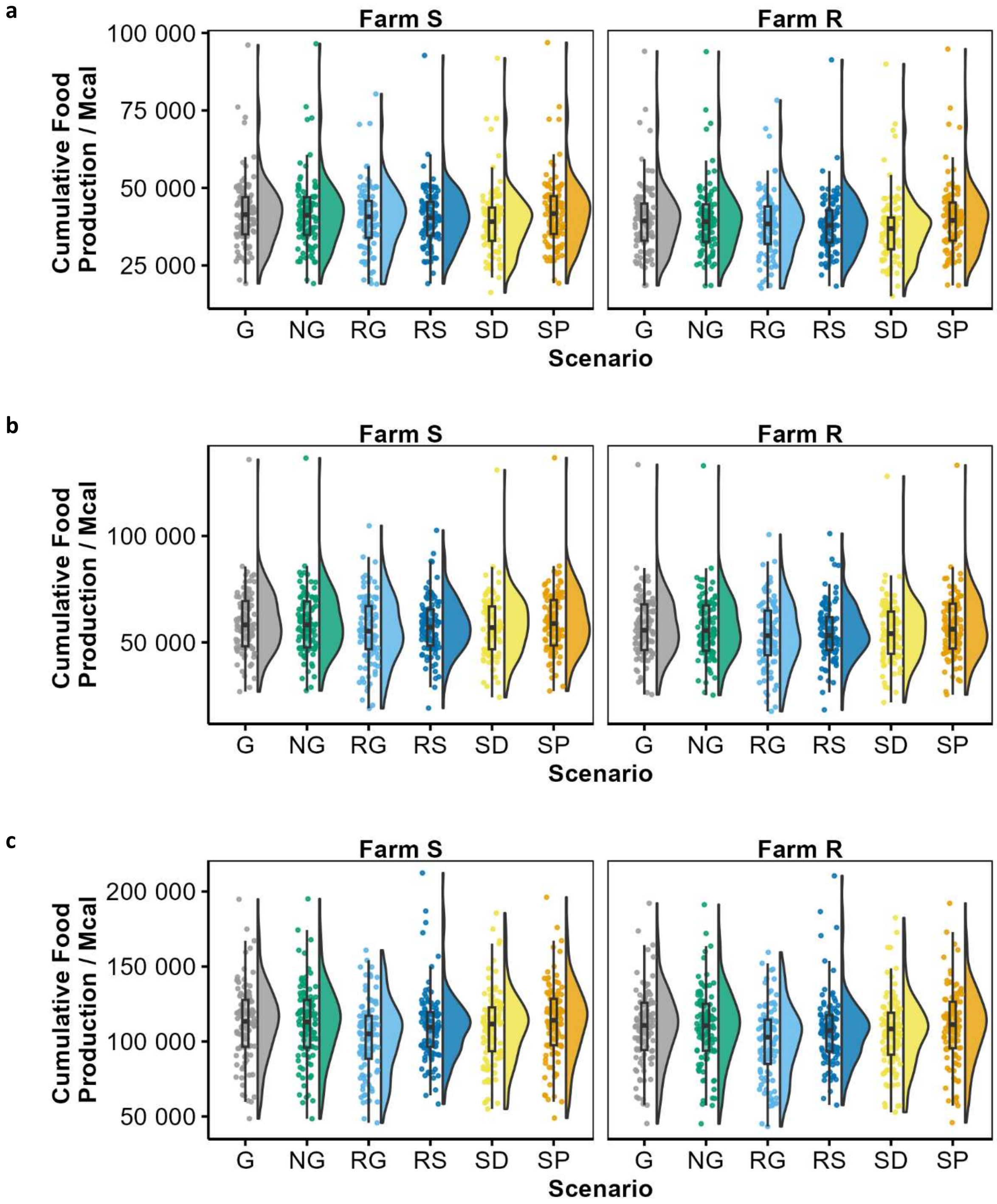

Figure 4: Cumulative food produced (Mcal) in each scenario after (a) 3, (b) 5, and (c) 10 years of simulation. Farm S has a weed community dominated by Poa annua and Farm R has a weed community dominated by Alopecurus

myosuroides. Scenarios are: glyphosate (G), no glyphosate (NG), increased frequency of grass leys (RG), increased frequency of spring crops (RS), delayed drilling of winter wheat crops by 3 weeks (SD) and, switch from minimum tillage to ploughing (SP).

Supplementary Results 4: Profit

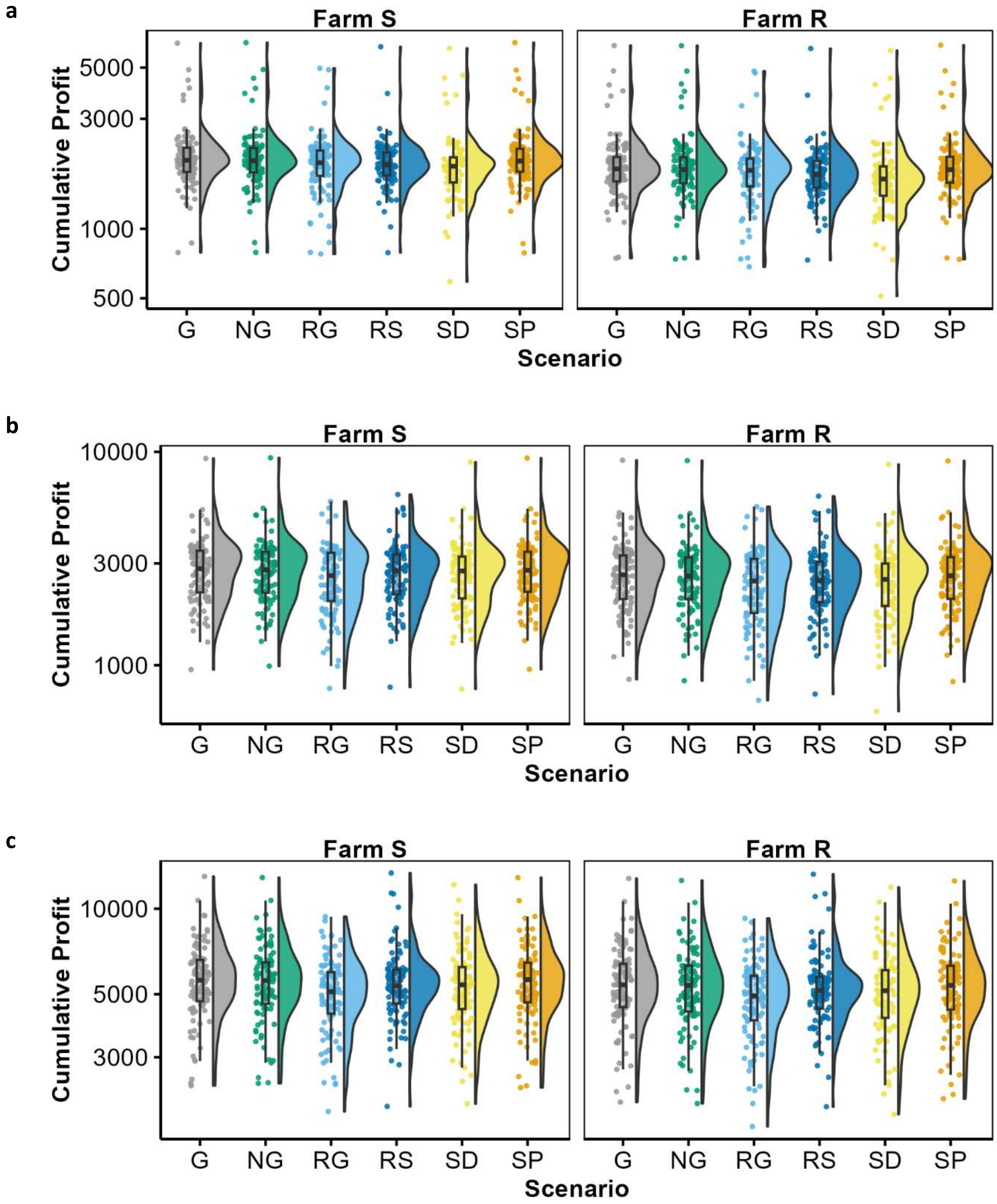

Figure 5: Cumulative profit made in each scenario after (a) 3, (b) 5, and (c) 10 years of simulation. Farm S has a weed community dominated by Poa annua and Farm R has a weed community dominated by Alopecurus myosuroides.

Scenarios are: glyphosate (G), no glyphosate (NG), increased frequency of grass leys (RG), increased frequency of spring crops (RS), delayed drilling of winter wheat crops by 3 weeks (SD) and, switch from minimum tillage to ploughing (SP).

Supplementary Results 5: EIQ

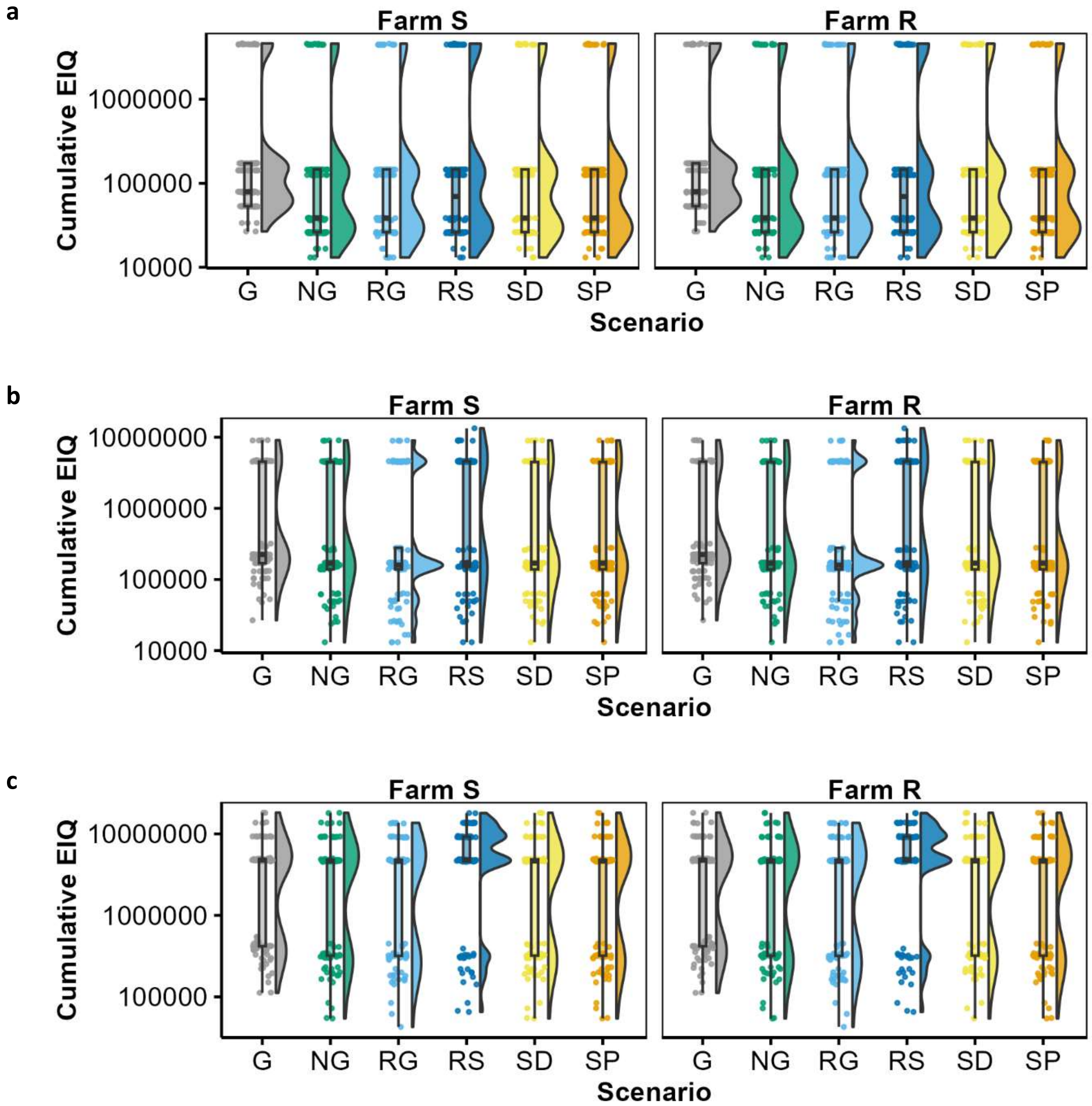

Figure 6: Total EIQ in each scenario after (a) 3, (b) 5, and (c) 10 years of simulation. Farm S has a weed community dominated by Poa annua and Farm R has a weed community dominated by Alopecurus myosuroides. Scenarios are: glyphosate (G), no glyphosate (NG), increased frequency of grass leys (RG), increased frequency of spring crops (RS), delayed drilling of winter wheat crops by 3 weeks (SD) and, switch from minimum tillage to ploughing (SP).

Supplementary Results 6: Crop Yields

In some cases the following figures show all levels to be not-significantly different according to Tukey tests despite a significant effect of the main effect in the lmm and a separation of the predicted means by an amount greater than the LSD. This is due to the lack of data at this scale and so estimation of the errors and LSDs may be inaccurate.

Spring Barley

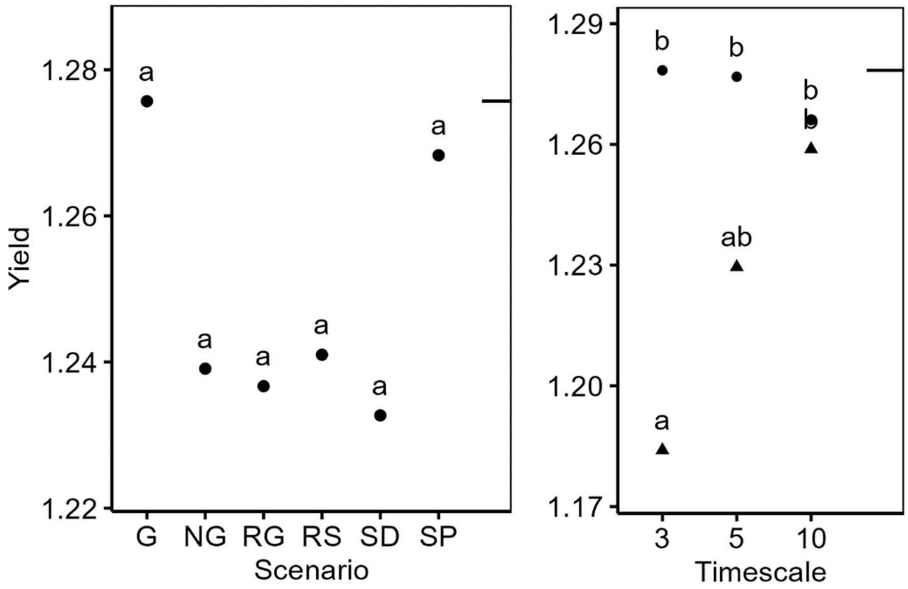

Figure 7: Predicted Spring Barley Yield (t ha^{-1}) from a linear model. Significant model terms were Scenario (P<0.001), Farm (P<0.001), and Farm:Timescale (P<0.001). Predictions are classified by the main effects of the Scenario and the interaction between the Farm and the Simulation year. Predictions are averaged over all levels of other terms included in the model. The error bar shows the approximate average LSD. Means within a single panel labelled with different letters indicates they are significantly different (P<0.05, post-hoc Tukey test).

Winter Barley

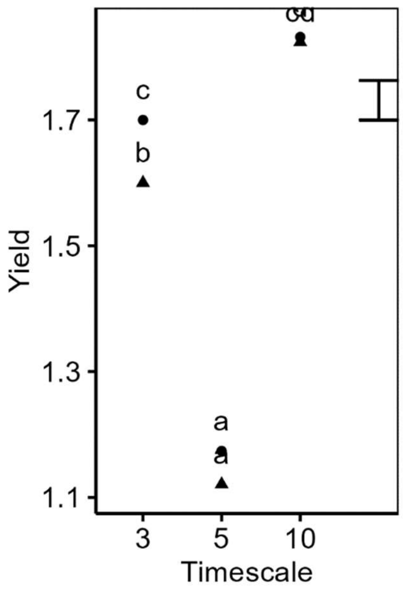

Figure 8: Predicted Spring Barley Yield (t ha^{-1}) from a linear model. Significant model terms were Farm (P<0.001), and Timescale (P<0.001) and Farm:Timescale (P<0.001). Predictions are classified by the main effects of the Scenario and the interaction between the Farm and the Simulation year. Predictions are averaged over all levels of other terms included in the model. The error bar shows the approximate average LSD. Means within a single panel labelled with different letters indicates they are significantly different (P<0.05, post-hoc Tukey test).

Beans

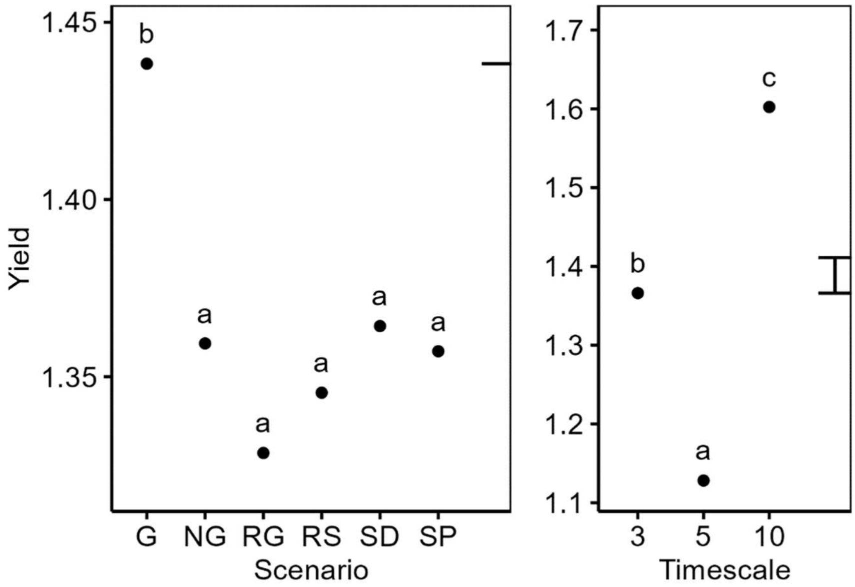

Figure 9: Predicted Bean Yield (t ha^{-1}) from a linear model. Significant model terms were Scenario (P<0.001), and Timescale (P<0.001). Predictions are classified by the main effects of the Scenario and the interaction between the Farm and the Simulation year. Predictions are averaged over all levels of other terms included in the model. The error bar shows the approximate average LSD. Means within a single panel labelled with different letters indicates they are significantly different (P<0.05, post-hoc Tukey test).

Maize

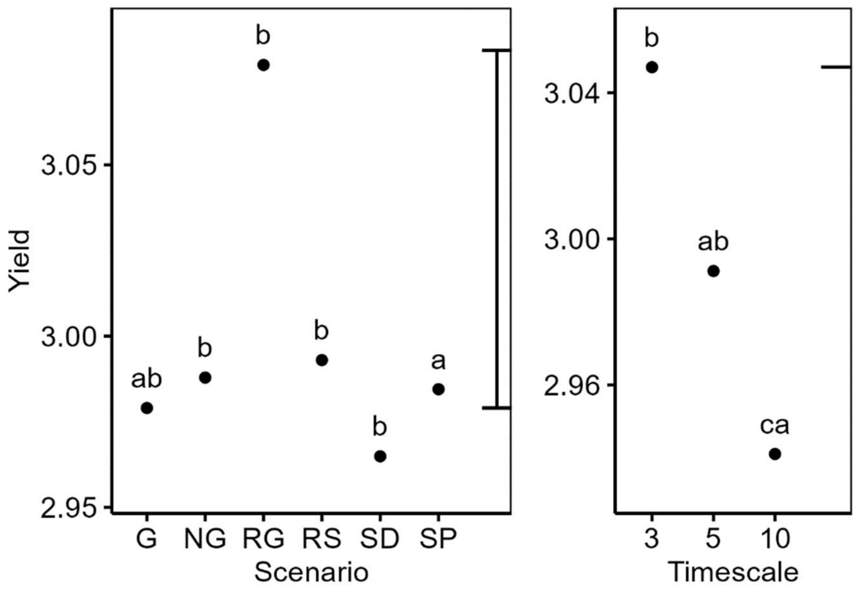

Figure 10: Predicted Maize Yield (t ha^{-1}) from a linear model. Significant model terms were Scenario (P<0.001), and Timescale (P<0.001). Predictions are classified by the main effects of the Scenario and the interaction between the Farm and the Simulation year. Predictions are averaged over all levels of other terms included in the model. The error bar shows the approximate average LSD. Means within a single panel labelled with different letters indicates they are significantly different (P<0.05, post-hoc Tukey test).

Oilseed Rape

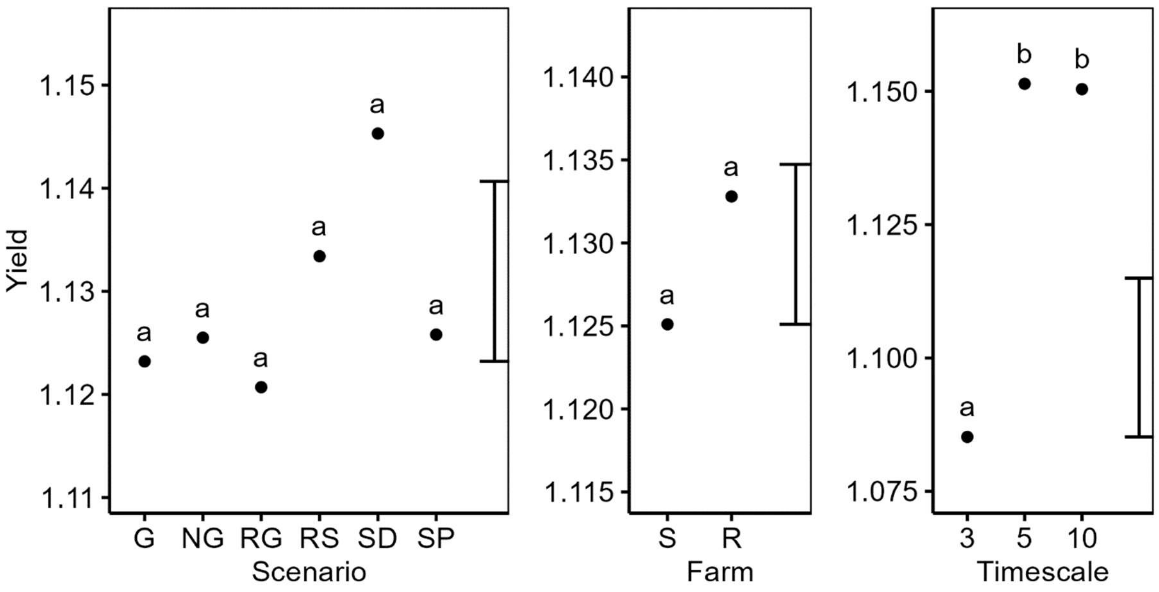

Figure 11: Predicted oilseed rape Yield (t ha^{-1}) from a linear model. Significant model terms were Scenario (P<0.001), Farm (P<0.001) and Timescale (P<0.001). Predictions are classified by the main effects of the Scenario and the interaction between the Farm and the Simulation year. Predictions are averaged over all levels of other terms included in the model. The error bar shows the approximate average LSD. Means within a single panel labelled with different letters indicates they are significantly different (P<0.05, post-hoc Tukey test).

Sugar Beet

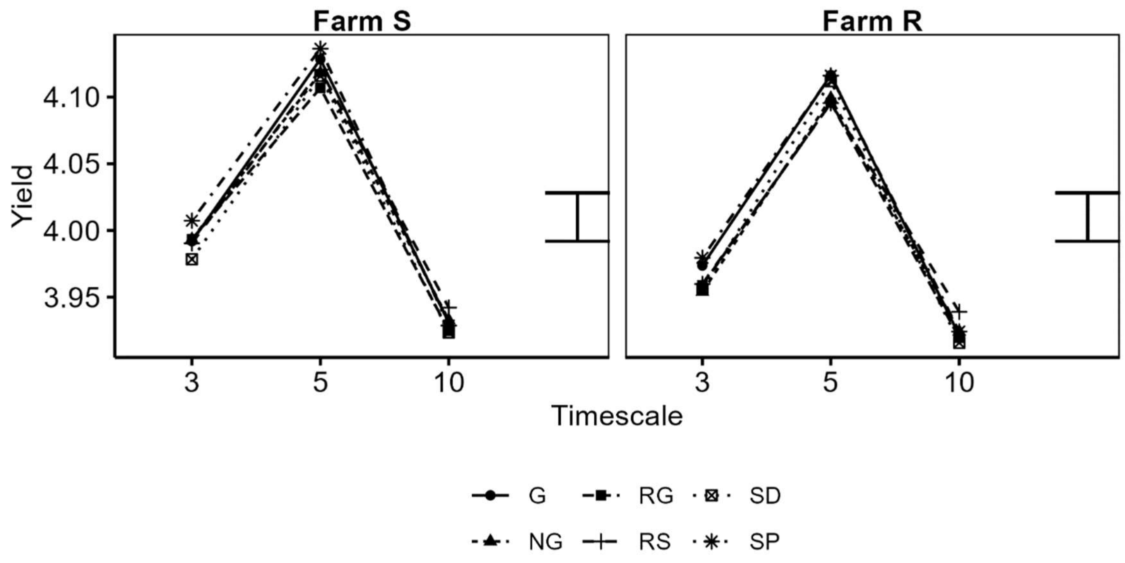

Figure 12: Predicted Sugarbeet Yield (t ha^{-1}*) from a linear model. Significant model terms were Scenario (P<0.001), Farm (P<0.001) and Timescale (P<0.001), Scenario:Timescale (P<0.05) and Farm:Timescale(P<0.001). Predictions are classified by the main effects of the Scenario and the interaction between the Farm and the Simulation year. Predictions are averaged over all levels of other terms included in the model. The error bar shows the approximate average LSD.*

Winter Wheat

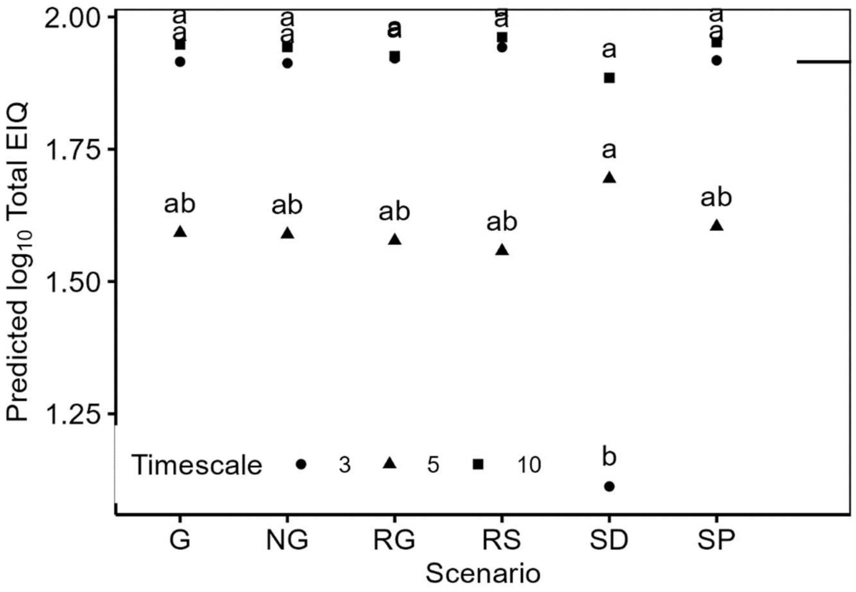

Figure 13: Predicted winter wheat Yield (t ha^{-1}) from a linear model. Significant model terms were Scenario (P<0.001), Timescale (P<0.001), Scenario:Timescale (P<0.001). Predictions are classified by the main effects of the Scenario and the interaction between the Farm and the Simulation year. Predictions are averaged over all levels of other terms included in the model. The error bar shows the approximate average LSD.

Spring Wheat

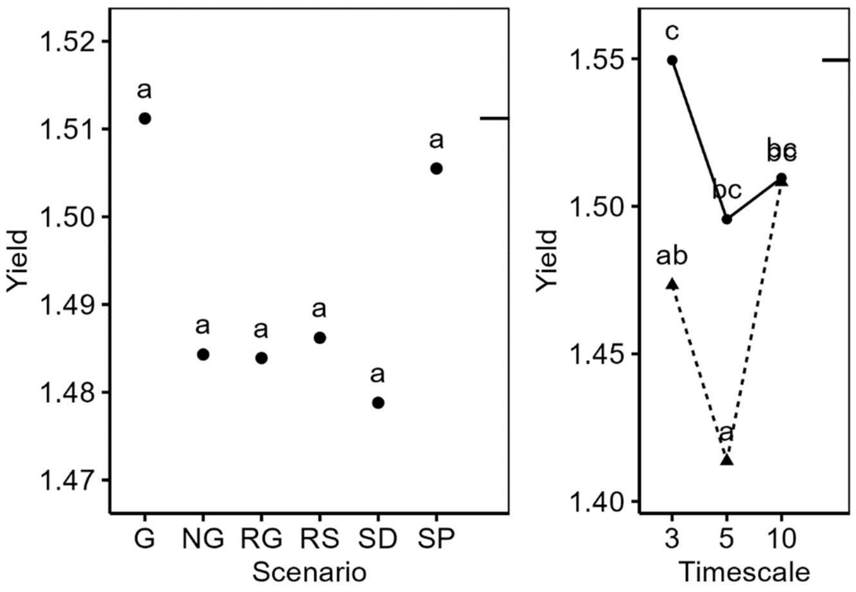

Figure 14: Predicted spring wheat Yield (t ha^{-1}) from a linear model. Significant model terms were Scenario (P<0.001), Timescale (P<0.001), Scenario:Timescale (P<0.001). Predictions are classified by the main effects of the Scenario and the interaction between the Farm and the Simulation year. Predictions are averaged over all levels of other terms included in the model. The error bar shows the approximate average LSD.
